# Therapy of Mucormycosis

**DOI:** 10.3390/jof4030090

**Published:** 2018-07-31

**Authors:** Nikolaos V. Sipsas, Maria N. Gamaletsou, Amalia Anastasopoulou, Dimitrios P. Kontoyiannis

**Affiliations:** 1 Pathophysiology Department, Medical School, National and Kapodistrian University of Athens, Mikras Asias 75, 115 27 Athens, Greece; nsipsas@med.uoa.gr (N.V.S.); magama@med.uoa.gr (M.N.G.); amanastasop@yahoo.gr (A.A.); 2 Department of Infectious Diseases, Infection Control and Employee Health, The University of Texas MD Anderson Cancer Center, 1515 Holcombe Blvd, Houston, TX 77030, USA

**Keywords:** mucormycosis, treatment, amphotericin B, posaconazole, isavuconazole, surgery, leukemia, stem cell transplantation

## Abstract

Despite the recent introduction of mold-active agents (posaconazole and isavuconazole), in addition to amphotericin B products, to our armamentarium against mucormycosis, many uncertainties remain for the management of this uncommon opportunistic infection, as there are no data from prospective randomized clinical trials to guide therapy. In this mini-review, we present the current status of treatment options. In view of the heterogeneity of the disease (different types of affected hosts, sites of infection, and infecting *Mucorales*), mucormycosis management requires an individualized management plan that takes into account the net state of immunosuppression of the host, including comorbidities, certainty of diagnosis, site of infection, and antifungal pharmacological properties.

## 1. Introduction

The term “mucormycoses” is used to describe a spectrum of chronic, subacute, and frequently rapidly progressing infections caused by fungi of the *Mucorales* order of the class of *Zygomycetes* [1]. Clinical presentations of mucormycosis are variable and include sinusitis (pansinusitis, rhino-orbital or rhino-cerebral), pulmonary, cutaneous, gastrointestinal, disseminated, and other uncommon presentations [2]. The most common agents causing mucormycosis are *Rhizopus* spp., *Mucor* spp., *Rhizomucor*, and *Leichtheimia* spp. Other genera less commonly implicated in infection include *Cunninghamella*, *Saksenaea*, and *Apophysomyces* [3]. These organisms are ubiquitous in nature as they can be found in decaying organic substrates and soil [4]. *Mucorales* are growing rapidly and they are releasing large numbers of airborne spores. Humans are exposed to those spores on a daily basis, but the intact immune system does not allow development of infection. Therefore, with the exception of victims of major natural disasters, the disease affects mainly immunocompromised patients with severe underlying diseases, such as hematologic malignancies (HM), solid organ (SOT) or hematopoietic stem cell transplantation (HSCT), uncontrolled diabetes mellitus, severe trauma, or burns [5,6,7,8,9,10,11,12,13,14,15,16,17,18]. Although mucormycosis represents a rare disease, its consequences are devastating, since it is associated with unacceptably high mortality rates, ranging from 20–50% if localised, up to 70–90% in cases of disseminated disease [2,19,20]. In heavily immunocompromised patients, *Mucorales* have a tropism for angioinvasion, resulting in dissemination, tissue infarction and necrosis. In contrast, cutaneous disease rarely disseminates and is associated with better outcomes [21,22].

In this article, we will discuss the current status of treatment options, as well as recent data concerning the management of this disease, that have emerged from clinical, in vitro, and in vivo studies.

## 2. Treatment

### 2.1. Difficulties in Evaluating Treatment Strategies in Mucormycosis

It is difficult to have robust data for the treatment of mucormycosis, because it is a rare disease, characterised by heterogeneity of hosts and sites of infection, as well as the multitude of offending *Mucorales*; therefore, no prospective, randomised clinical studies exist. Information regarding the current approach to treatment is based on single-institution, retrospective studies including a limited number of patients with significant variability in their presentation and risk factors, registries with methodological flaws, and “expert opinions”. Suboptimal diagnosis based on tissue culture and/or histology misses cases and biases treatment experience towards culture and/or histology-positive cases (that is with high burden of disease) or towards selected patient groups, where tissue is more readily accessible, such as sinusitis or trauma patients. The low quality of data is reflected on the recently published European guidelines [23,24], where the grading of evidence is suboptimal and therefore, their usefulness in the clinical practice is questionable.

### 2.2. Principles of Treatment of Mucormycosis

**Figure 1:**
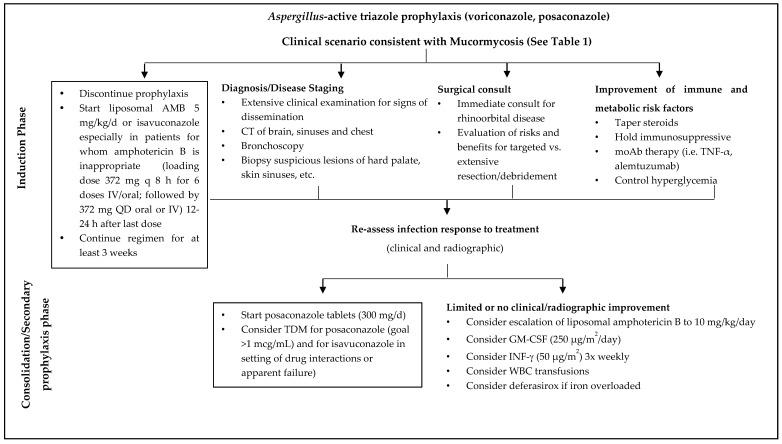
Algorithm for Mucormycosis Treatment. AMB: amphotericin B; CT: computed tomography scan; moAb: monoclonal antibodies; TDM: therapeutic drug monitoring; GM-CSF: granulocyte-macrophage colony stimulating factor; INF-γ: interferon—gamma; WBC: white blood cells.

The management of mucormycosis is based on multiple interventions occurring simultaneously, or with different timing and intensity. The basic principles of mucormycosis treatment include risk stratification for severity of the diseases, and intense attempts for early, clinical and laboratory diagnosis; timely initiation of an effective antifungal therapy (monotherapy or combination therapy) along with aggressive surgical debridement of necrotic lesions; reverse of immunosuppression (discontinuation of chemotherapy and increase of neutrophils), and when feasible control of the underlying medical condition. Early diagnosis and prompt therapeutic intervention may prevent progressive tissue invasion and its sequelae, may also reduce the need for extensive surgery and subsequent deformity, and may improve survival [25]. In a study of 70 patients with hematological malignancies and mucormycosis, delayed antifungal therapy for ≥6 days after diagnosis resulted in a 2-fold increase in mortality rate, compared with early treatment (82.9% vs. 48.6%) [26]. No treatment is not an option, as untreated mucormycosis is universally fatal. Roden et al., in an analysis of 929 published cases of mucormycosis [2] demonstrated by multivariate analysis that antifungal therapy and surgery were significantly associated with improved survival rates (69%), while death was almost inevitable (97%) for patients who received no treatment at all. Figure 1 depicts our suggested approach to management of mucormycosis.

### 2.3. Antifungal Agents for Mucormycosis

Only amphotericin B (AMB) and its lipid formulations, and recently isavuconazole [27] have been studied as first-line therapy for mucormycosis. On the contrary, posaconazole has been mainly studied as salvage therapy [23,24]. The efficacy of these agents is based on limited clinical data and on preclinical in vitro/in vivo data, showing activity against *Mucorales*. It should be underlined though, that no validated minimum inhibitory concentration (MIC) breakpoints exist for any of these agents [23,24,28].

#### 2.3.1. Lipid Formulations of Amphotericin B

AMB is considered the drug of choice for primary treatment of mucormycosis. The efficacy of AMB has been shown in both laboratory (in vitro and in vivo) and clinical studies [29]. Although interpretive breakpoints to AMB have not been determined, high in vitro MICs to AMB have been observed in clinical isolates of *Cunninghamella* species [30]. However, in a small study of non-*Aspergillus* invasive mould infections, an MIC for amphotericin B of ≤0.5 µg/mL was significantly associated with better 6-week outcomes [31]. Lipid formulations of amphotericin B (liposomal AMB, LAMB; and AMB lipid complex, ABLC) have better therapeutic index than the conventional amphotericin B deoxycholate and are considered as the first-line therapy of mucormycosis [23,24,32,33].

As with many antifungal agents and mycoses, the optimal dosage for AMB and its formulations against mucormycosis is still undetermined. The standard daily dose of LAMB and ABLC suggested by current guidelines is 5 mg/kg/day [23,24]. The in vitro activity of AMB against *Mucorales* is highly variable [28,34]. Recently, researchers reported that among 524 clinical *Mucorales* isolates, the epidemiologic cut-off values (ECVs) ≥97.5% for amphotericin B were rather high: 2 µg/mL for *L. corymbifera*, 2 µg/mL for *M. circinelloides*, 4 µg/mL for *R. arrhizus*, and 2 µg/mL for *R. microspores* [34]. These relatively high AΜB MIC values support the use of higher daily dose of AMB to achieve clearance of *Mucorales* from tissues. Indeed, in a neutropenic murine model of pulmonary mucormycosis, the efficacy of liposomal AMB was dose-dependent: a dose of 10 mg/kg/day has been proved to be more effective in reducing fungal burden compared to 5 or 1 mg/kg/day [35]. Based on these in vitro and in vivo data, researchers proposed to treat mucormycosis with high dose LAMB (>5 mg/kg/day) [36]. Yet, this recommendation has not been supported by the findings of a subsequent clinical study. The French Mycoses Study Group conducted a phase I–II prospective, multicenter, pilot trial on the efficacy and safety of high-dose (10 mg/kg/day) LAMB monotherapy (AmBizygo study) for the treatment of mucormycosis. The study included 40 patients, the majority of them with underlying hematological malignancy and/or HSCT. Surgery and debridement has been performed in 71% of the patients before initiation of antifungal therapy. Compared to historical controls receiving the standard dose of 5 mg/kg/day, no improvements in mortality and response rates (38 and 36% respectively) was seen at 12 weeks of treatment. On the other hand, high dose L-AMB was associated with increased nephrotoxicity and electrolyte derangements. Characteristically, doubling of the baseline serum creatinine levels has been observed in 40% of the patients, dictating dose reduction [37]. Although dosages beyond 5 mg/kg/day have not been proved to be more efficacious for mucormycosis, they may be considered on an individual basis, especially when there is CNS or osteoarticular involvement [24].

#### 2.3.2. New Triazoles

Triazoles act by depleting ergosterol from the fungal cell membrane. Among triazole antifungals, fluconazole, itraconazole, and voriconazole have little or no activity against *Mucorales*. Newer triazoles, namely posaconazole and isavuconazole, have better in vitro activity against *Mucorales* and clinical data supporting their use in mucormycosis [27,38].

##### Posaconazole

Posaconazole has variable in vitro activity against *Mucorales*, which is species-dependent [30]. A study of 131 clinical isolates showed that the median MICs of posaconazole for various *Mucorales* species varied widely between 1.0 and 8.0 µg/mL [28]. In laboratory animal studies, experimental infections produced by *Mucor* spp. were most responsive to posaconazole, while those caused by *Rhizopus* spp. were usually non-responsive [39,40,41]. Lewis et al. have shown in an immunosuppressed murine model of pulmonary mucormycosis, that a posaconazole serum concentration higher than 4000 µg/mL is needed to suppress the growth of *Rhizopus* spp. with an MIC of 2 µg/mL [39]. These data raise concerns on the clinical efficacy of posaconazole, at least in the current standard dose of 300 mg/day of extended release tablets, as *Rhizopus* is among the most common agents causing mucormycosis.

Clinical studies on the efficacy of posaconazole for mucormycosis are scarce. Early case reports and case series reported that posaconazole could be an option as salvage therapy in patients unresponsive or intolerant to LAMB [42,43]. In a subsequent open-label trial including 24 patients, the success rate of salvage therapy with posaconazole oral suspension (800 mg in 4 divided doses) was 70%. The drug was well-tolerated with only minor gastrointestinal side-effects [44]. In another retrospective study of posaconazole oral suspension as salvage therapy in 91 patients with refractory mucormycosis, the response rate was 61%, and in the subgroup of patients with the pulmonary form of mucormycosis 65%. An additional 21% of subjects had stable disease at the end of 12 weeks of treatment [45].

Until recently, posaconazole was available only as oral suspension, administered three or four times daily, with food (preferably a high-fat meal) or with an acidic carbonated beverage, in order to enhance bioavailability. These food requirements make difficult the use of the oral solution in critically ill patients, as they might not be able to eat or they might be nauseous [46,47,48]. Therefore, absorption of posaconazole oral suspension was often suboptimal leading to therapeutic failures [48]. To overcome the pharmacokinetic limitations of the oral solution a gastro-resistant tablet and an intravenous (IV) solution has been developed [49]. The advantages of the tablet formulation over the suspension include better bioavailability allowing once-daily dosage, no food requirements, absorption unaffected by changes in gastric pH or motility; less interpatient variability and more predictable plasma concentrations than the suspension [50]. Despite improved pharmacokinetics, therapeutic drug monitoring (TDM) is suggested for the tablets as it is the case for the suspension formulation [51]. For the rare clinical scenario where patients are unable to take oral drugs, an intravenous formulation of posaconazole containing β-cyclodextrin had been developed, with excellent pharmacokinetic properties [52]. The role of the tablet and IV formulations in the treatment of mucormycosis has not been defined. Currently, posaconazole (oral suspension 400 mg × 2/day when taken with meals, or 200 mg × 4/day if not taken with meals) may be considered as salvage treatment of mucormycosis. First–line treatment with posaconazole is considered only in cases when treatment with AMB is absolutely contraindicated [23,24], although isavuconazole might be a better option in this situation, as primary treatment data exist only for this newer azole [27].

##### Isavuconazole

Isavuconazole is a new broad-spectrum triazole and is the biologically active agent of the prodrug isavuconazonium sulfate. It is approved in the United States for the treatment of mucormycosis, and in Europe for the treatment of mucormycosis when amphotericin B is not feasible [53]. It is available in both intravenous and oral formulations and it is administered with a loading dose of 200 mg three times a day for two days and 200 mg daily thereafter. Isavuconazole has many pharmacokinetic and safety advantages compared to other azoles, including linear pharmacokinetics and thus no need for TDM; less drug–drug interactions; less toxicity, especially hepatotoxicity, skin and ocular side-effects, or QT prolongation; no nephrotoxic cyclodextrin in the IV formulation; no need for dose adjustment in kidney, liver failure or obesity; and excellent oral bioavailability with no food requirements [53,54,55,56,57,58].

As it is the case with posaconazole, isavuconazole has variable, species-dependent, in vitro activity against *Mucorales* [56,59]. It should be noted that the MIC values of isavuconazole for *Mucorales* are 2- to 4-fold higher compared to those of posaconazole, and this should be taken into account in clinical practice [59]. In the neutropenic mouse model of mucormycosis due to *Rhizopus*, isavuconazole had comparable efficacy to high-dose L-AmB in reduction of tissue fungal burden, in both the lung and the brain, and provided a survival benefit at 21 days of treatment [60]. Several case reports have shown that isavuconazole could be used successfully as salvage therapy for mucormycosis, in heavily immunosuppressed patients, including cases of posaconazole failure [61,62,63].

VITAL study, a phase 3, single-arm, open-label, noncomparative trial was designed to evaluate the safety and efficacy of isavuconazole in the treatment of invasive aspergillosis in patients with renal impairment or in patients with invasive fungal disease caused by rare moulds, yeasts, or dimorphic fungi. The study was done in 34 centres worldwide and in accordance with the US Food and Drug Administration’s guidance for comparators in studies of rare diseases, the VITAL study used as controls matched cases treated with LAMB from the FungiScope registry [27,64].

Among the study population, 37 patients had proven or probable mucormycosis as per the European Organisation for Research and Treatment of Cancer/Mycoses Study Group (EORTC/MSG) criteria. The Data Review Committee (DRC) classified patients to those receiving isavuconazole as primary treatment (21 patients), and as salvage therapy for mucormycosis refractory to prior antifungal therapy (11 patients) or due to intolerance to other antifungals (*n* = 5). Among patients, 13 (35.1%) were HSCT recipients, and 3 (8.1%) were SOT recipients. The most common site of involvement were lungs (*n* = 22); in half of cases there was concurrent involvement of other organs. Other sites were sinus (*n* = 16), eye (*n* = 7), central nervous system (*n* = 6), and disseminated disease (*n* = 11). The species distribution included *Mucor* spp. (*n* = 20), *Rhizopus* spp. (*n* = 9), *Rhizomucor* spp. (*n* = 5), *Lichtheimia* spp. (*n* = 2), and one isolate of *Cunninghamella* spp. Isavuconazole MIC values ranged from 0.25 to >16 µg/mL. By treatment day 42, 4 (11%) of 37 patients had a partial response to isavuconazole treatment, 16/37 (43%) patients had stable disease and 1/37 (3%) had disease progression. All-cause mortality through day 42 was 38% (14/37 patients); 33% (7/21) for patients who received isavuconazole as primary therapy, 46% (5/11) for patients with mucormycosis refractory to other antifungal therapy, and 40% (2/5) for patients who were intolerant of other antifungal drugs. The median treatment duration was 102 days for patients classified as primary, 33 days for refractory, and 85 days for intolerant. Notably, in 8 additional patients with mixed invasive fungal diseases that included a *Mucorales* infection, all-cause mortality by day 42 was 25% (2/8 patients) and by day 84 was 38% (3/8 patients). For the case-control analysis, 33 patients treated with AMB in the FungiScope registry were used as controls. Survival probability through day 84 was similar between isavuconazole patients (57%) and AMB historical controls (50%). Overall, 24 patients discontinued isavuconazole treatment. Main reasons for discontinuation were death (30%), adverse events including acute liver injury of unclear cause (16%), and non-compliance (11%). Gastrointestinal symptoms were the most common side-effects. No relation between trough serum levels of isavuconazole, fungal MICs, and outcomes was detected [27].

The VITAL study had many limitations. Most patients given isavuconazole were pre-exposed to AMB, with an unknown effect on outcomes. As the number of patients was small, the study was underpowered to detect clinically meaningful differences between isavuconazole and LAMB. Also, there are methodological issues, as matching of patients enrolled in prospective studies with historical controls underestimates known and unknown confounding factors that affect outcomes.

Of major concern are recently emerging studies reporting cases of breakthrough mucormycosis and other IFIs in patients receiving isavuconazole as prophylaxis or treatment [65,66]. In a trial of intravenous isavuconazole prophylaxis in acute myeloid leukemia patients, 18% (2/11) of patients had possible breakthrough fungal infection [67]. In 2016, Dadwal et al. reported 6 (4.6%) episodes of breakthrough IFIs among 131 patients with hematologic malignancy receiving isavuconazole more than seven days [68]. In another series of 100 leukemic patients treated with isavuconazole, thirteen (13%) patients developed proven (*n* = 11), probable (*n* = 2), or possible (*n* = 7) breakthrough IFIs [65]. The majority (70%) of these patients were profoundly neutropenic. Half of the microbiologically documented lung infections in that study were caused by *Mucorales* [65]. In another study, five cases of breakthrough IFIs have been reported among patients with hematological malignancies receiving isavuconazole as prophylaxis [66]. The reported fungal species that caused breakthrough infection included *Aspergillus* spp., *Mucorales*, non-albicans *Candida* spp., and *Scedosporium apiospermum*. Such cases have not been reported in the regulatory studies of isavuconazole. These alarming reports suggest that selection of isavuconazole-resistant *Mucorales* and other fungi may occur during protracted isavuconazole administration, in the setting of profound immunosuppression and suboptimal isavuconazole trough levels, especially if we take into consideration the relatively high Isavuconazole MICs for *Mucorales* [65]. There is also the theoretical issue of cross-tolerance, where patients exposed to prolonged prophylaxis with posaconazole might develop breakthrough mucormucosis, which is resistant to subsequent treatment with isavuconazole [41,69].

##### Combination Therapy

Despite the lack of solid clinical data therapy of mucormycosis in heavily immunosuppressed patients with a combination of antifungals has become an increasingly common practice. The pros of such therapeutic approach are synergistic effect and broader coverage, and the cons possible antagonism, drug interactions, toxicity, and cost [70].

In vitro studies and in vivo animal model investigations have shown evidence of synergism between polyenes and echinocandins. Although inherently inactive against *Mucorales*, in vitro echinocandins are considered to have some in vivo effect, through degradation of the small amount of glucan on the cell wall of the fungus, with unmasking of immune epitopes, and facilitation of phagocytosis [71,72,73,74,75,76]. In one retrospective study among diabetic patients with rhino-orbital or rhino-cerebral mucormycosis, the combination of AMB + echinocandin was successful in 6 of 7 treated patients compared with only 7 of 22 patients treated with ABLC monotherapy (*p* = 0.02) [75]. However, in patients with hematological malignancies, the combination was not so successful. A recent, single-institution study of 106 HM patients with mucormycosis failed to show any benefit from combination treatment, compared to AMB monotherapy, in both unadjusted analysis, and after propensity-score-based adjustment for confounding factors [76].

Data on the efficacy of the AMB + triazole combination for the treatment of mucormycosis are contradictory. In vitro studies have shown synergy for the combination of a polyene and posaconazole, but in vivo studies in murine models of mucormycosis showed no benefit when the agents are used together [77,78]. Similarly, a study in neutropenic mice infected with *R. oryzae* found that amphotericin B and posaconazole dose combination did not prolong survival or decrease organ fungal burden to a greater extent than AMB monotherapy [79]. Human studies evaluating polyene-triazole combination for the treatment of mucormycosis are limited [76,80,81,82,83,84,85]. In one retrospective case series, 32 patients with hematological malignancies or aplastic anemia, and mucormycosis unresponsive to prior monotherapy (mainly with LAMB), were treated with combination of polyene and posaconazole [86]. After three months of treatment, 18 patients (56%) had clinical improvement and nine patients (28%) did not respond or died from progression of disease. The modest existing pre-clinical and clinical data do not support the use of combination therapy, with the possible exception of CNS mucormycosis, where a combination of high-dose LAMB and posaconazole or isavuconazole might be considered.

### 2.4. Surgery

Surgical resection of necrotic tissues is the core of mucormycosis therapy [23,24]. In pulmonary mucormycosis, surgical treatment along with appropriate systemic antifungal therapy has been shown to significantly improve survival compared to antifungal therapy alone [87,88]. Bouts of hemoptysis due to cavitation of lesions near hilar vessels is an indication for urgent resection of the lesion [87,89]. In certain cases of localised disease surgery might be curative. In patients with rhino-orbital mucormycosis, magnetic resonance imaging might have a role in staging the resectability of the lesions [90,91].

Similarly, surgical removal of infected tissues is of paramount importance in the treatment of rhino-orbital-cerebral disease [29,92]. It should be underlined however, that the effect of surgery on outcome is difficult to be defined, due to selection biases. An endoscopic approach is preferred over the open surgery in patients with early, limited disease, or with significant medical comorbidities [93,94]. Open surgeries are preferred for extensive disease, and include maxillectomy, orbital exenteration and/or craniofacial resection; yet, no survival benefit has been proved for such radical approach, especially in patients with limited life expectancy [70,92,95]. Researchers reported the outcome of surgical management in 22 patients with rhino-orbito-cerebral mucormycosis. Local control of the disease with wide and repeated surgical debridement (performed in 45% of patients) was associated with improved outcomes. Local control was obtained in 90% of the patients after radical surgery vs. 41.6% in patients who had limited surgery [96].

Finally, in a 10 year retrospectively study of 44 patients with hematologic malignancy and/or HSCT and invasive fungal sinusitis, due to *Mucorales* in 13 cases, showed that early endoscopic surgery did not improved overall outcome. The major risk factors for death, not fungal-related, were relapsed and/or refractory malignancy and protracted neutropenia [90].

### 2.5. Adjunctive Therapy

Reversal of immunosuppression is an important pillar of therapy for mucormycosis, along with surgery and appropriate early antifungal agents. Most patients who die of this disease have poor recovery of bone marrow function or require prolonged immunosuppressive therapy (such as those with GVHD). Therefore, any effort to reverse neutropenia in hematology patients should be made, by using hematopoietic growth factors, or in selected cases, by white cell transfusions. Patients with immunosuppression from corticosteroids, such as patients with autoimmune diseases should be tapered or transitioned to alternative non-steroidal therapy, if possible. Patients with HIV/AIDS should be started on anti-retroviral therapy, in order to restore their immunity. Aggressive glycemic control is paramount for patients with uncontrolled diabetes and/or ketoacidosis. Reversal of acidemia by administration of sodium bicarbonate is able to partially block the ability of *Rhizopus oryzae* to invade endothelial cells, and to restore host iron chelation and neutrophil function [97]. Researchers proposed iron chelators as a potential adjunctive therapy, by means of reducing the available iron and thus inhibiting the fungal growth. Preclinical data in a mice model showed increased survival when receiving deferasirox, a new iron chelator that lacks siderophore capability [98]. However, a subsequent prospective, randomized study in patients with hematologic malignancies showed that the combination of LAMB with deferasirox was associated with increased mortality [99]. Although administration of deferasirox seems to lack benefit in patients with hematologic malignancy and mucormycosis, it remains a plausible therapy in other high-risk patients, such as diabetic patients [24], especially those with ketoacidosis where the low pH increases the levels of unbound tissue iron, promoting thus the growth of *Mucorales*.

The increased oxygen pressure achieved with hyperbaric oxygen (HBO) treatment improve the functionality of neutrophils. Furthermore, HBO promotes the AMB action by reversing acidosis [100]. Finally, high oxygen pressure inhibits fungal growth and improves the rate of wound healing. Thus, treatment with HBO has been proposed as adjunct to surgical and antifungal therapy for mucormycosis, particularly in diabetic patients who have sinusitis, or in cutaneous mucormycosis [100,101,102,103,104,105,106,107]. In a review of 28 cases adjunctive HBO was beneficial in diabetic patients (94% survival), but not in patients with haematological malignancies or bone marrow transplants (33% survival; *p* 0.02). Prolonged courses of HBO were associated with a higher survival (100% survival; *p* 0.003), although this can be explained by “survival bias” (107). Yet, the lack of prospective studies and controls make the efficacy of the method debatable.

Immune-augmentation strategies, such as administration of granulocyte (macrophage) colony-stimulating factor or interferon-γ have been proposed as adjunct therapy, based on limited in vitro data and case-reports [108,109]. Granulocyte transfusions have also been tested with unclear success but with some risk for inflammatory lung injury [110]. Statins have shown in vitro and in vivo activity against *Rhizopus* spp., but reliable clinical data are lacking [111,112]. In a recent case report, an immunosuppressed, trauma patient with intractable mucormycosis was successfully treated with combination of interferon-γ and nivolumab [113], a monoclonal antibody that decreases programmed death-1 (PD-1) expression on T-cells. Interferon-γ restores monocyte function and has been used as rescue therapy for life-threatening fungal infections [114] while nivolumab binds to PD-1, blocks interaction with its ligands, and enhances PD-1 pathway-mediated inhibition of T-cell proliferation and cytokine production. Anti-PD-1 has shown activity in animal models of fungal sepsis [115]. Given the limited evidence, the relative benefit of adjunctive strategies must be balanced against the cost and potential for harm, on an individual patient basis.

### 2.6. Treatment Duration

There is no standard duration of treatment for mucormycosis. Decisions are made on an individual basis, and as a principle, antifungal therapy of mucormycosis is continued until resolution of all clinical, laboratory, and imaging signs and symptoms of infection and reversal of immunosuppression. Oral formulations of newer azoles with activity against *Mucorales*, such as posaconazole and isovuconazole have an important role in bridging the initial IV treatment of mucormycosis to long-term treatment [23]. In selected patients, positron emission tomography/computed tomography scan might have a role in making the distinction between radiographic signs of active disease and inactive scars, thus facilitating treatment discontinuation [36,116].

### 2.7. New Antifungal Agents with Activity against Mucorales

The number of therapeutic options for the treatment of mucormycosis is quite limited when compared with those available to treat bacterial or other fungal infections. Indeed, only two classes of molecules, polyenes and azoles, are currently used in clinical practice. Unlike invasive candidiasis and aspergillosis, the majority of hematology patients who develop mucormycosis still die from their infection despite the administration of systemic antifungal therapy, emphasizing the need for new antifungal drugs with activity against *Mucorales*. Yet, it is difficult to find unique targets for *Mucorales*. Recent genome sequencing of *Rhizopus oryzae* revealed evidence of a whole-genome duplication event during its evolution [117], offering a remarkable genetic plasticity to this pathogen and explaining its relative resistance to multiple antifungal classes.

The investigational agent VT-1161 is a novel inhibitor of the fungal CYP 51 and has in vitro activity against *Mucorales*, including *Rhizopus oryzae*, *Lichtheimia*, and *Cunninghamella* [118]. In a murine model, it was shown to prolong survival of neutropenic mice with mycormucosis due to *Rhizopus oryzae* when given therapeutically or as prophylaxis [119,120]. APX001A (formerly E1210) is an antifungal agent that targets Gwt1, an early step in the conserved glycosylphosphotidyl inositol (GPI) post-translational modification pathway of surface proteins in eukaryotic cells. Although it has modest in vitro activity against other *Mucorales* [121], in a recent study, it protected immunosuppressed mice from *Rhizopus delemar* infection [122]. APX001A has entered phase I clinical trials. Finally, another novel agent, haemofungin inhibits in vitro the growth of several fungi including *Rhizopus* [123].

## 3. Conclusions/Future Directions

Existing data on the therapy of mucormycosis are not very helpful to clinicians managing this dreadful infection. Case reports and single-institution retrospective case series should be interpreted with caution due to multiple inevitable biases. Multiple controversies remain (Table 1), as numerous host-related, microbiological, surgical, and pharmacological factors affect the outcomes and lead to highly individualized scenarios for the management of mucormycosis. Discovery of novel drug targets and design of “pragmatic” prospective, multicentre studies and registries are of paramount importance for developing more efficacious treatment strategies.

## Figures and Tables

**Table 1:** Controversies in the management of Mucormycosis (MCR).

What is the role of PCR-based platforms (serum, BAL, tissues) for the diagnosis of MCR?
What is the role of immunohistochemistry for the tissue diagnosis of MCR?
What is the best diagnostic approach for pulmonary MCR (BAL versus CT-guided FNA versus VATS)?
What is the role of in vitro susceptibility testing for *Mucorales* isolates in guiding MCR management?
What is the role of combination AMB/triazole versus switching to AMB in patients with triazole-associated breakthrough MCR?
What is the role of switching triazoles as monotherapy in isavuconazole- or posaconazole-associated breakthrough MCR?
What is the role of increasing the dose of *Mucorales*-active triazoles as monotherapy (e.g., posaconazole) in MCR?
What is the optimal timing of de-escalation to a triazole after the onset of pre-emptive liposomal AMB—based treatment of breakthrough invasive *Mucorales* infection?
What is the optimal antifungal management in patients with MCR and baseline liver and/or renal dysfunction?
What is the role of immune restoration via corticosteroid tapering in MCR outcome?
What is the role of metabolic control (e.g., correction of ketoacidosis in MCR patients with diabetes) in the management and outcome of MCR?
What is the role of adjunct immunotherapy (e.g., GM-CSF ± interferon, or check point inhibitors) in the MCR management?
What is the role of surgical de-bulking of isolated pulmonary (or central nervous system) lesions in the setting of MCR?
What is the optimal timing and extent of surgical debridement in isolated sinus MCR? How often one needs to evaluate the need for repeat debridement in sinusitis?
What is the optimal timing of resuming chemotherapy after diagnosis of MCR?
Is prior MCR a relative or an absolute contraindication for subsequent HSCT or solid organ transplantation?
How often is an ENT outpatient re-evaluation needed in patients with history of MCR of the sinuses?
Is there a role of PET/CT in long term evaluation and follow up of patients with MCR?

Abbreviations: AMB, amphotericin B; BAL, bronchoalveolar lavage fluid; GM-CSF, glanulocyte macrophage colony stimulating factor; CT, computed tomography; FNA, fine needle aspiration; HSCT, hematopoietic stem cell transplantation; PCR, polymerase chain reaction; VATS, video-assisted thoracoscopic surgery; ENT, ears nose and throat; PET/CT, positron emission tomography/computed tomography.
